# Multiple mediation of the association between childhood emotional abuse and adult obesity by anxiety and bulimia – a sample from bariatric surgery candidates and healthy controls

**DOI:** 10.1186/s12889-024-18015-w

**Published:** 2024-03-01

**Authors:** Hongwei Zhang, Ziqi Liu, Hui Zheng, Ting Xu, Lin Liu, Tao Xu, Ti-Fei Yuan, Xiaodong Han

**Affiliations:** 1 Department of Bariatric & Metabolic Surgery, Shanghai Sixth People’s Hospital Affiliated to Shanghai Jiao Tong University School of Medicine, Shanghai, China; 2 Shanghai Key Laboratory of Psychotic Disorders, Brain Health Institute, National Center for Mental Disorders, Shanghai Mental Health Center, Shanghai Jiao Tong University School of Medicine, Shanghai, China; 3 Department of Psychology, Anhui Provincial Children’s Hospital, Children’s Hospital of Fudan University Anhui Hospital, National Children’s Regional Medical Center, Hefei, China; 4 Department of Anesthesiology, Shanghai Sixth People’s Hospital Affiliated to Shanghai Jiao Tong University School of Medicine, Shanghai, China; 5 Co-innovation Center of Neuroregeneration, Nantong University, Nantong, Jiangsu China; 6 Department of Endocrinology and Metabolism, Shanghai Sixth People’s Hospital Affiliated to Shanghai Jiao Tong University School of Medicine, Shanghai, China

**Keywords:** Obesity, Childhood emotional abuse, Anxiety, Bulimia, Multiple mediation model

## Abstract

Bulimia, which means a person has episodes of eating a very large amount of food (bingeing) during which the person feels a loss of control over their eating, is the most primitive reason for being overweight and obese. The extended literature has indicated that childhood emotional abuse has a close relationship with adverse mood states, bulimia, and obesity. To comprehensively understand the potential links among these factors, we evaluated a multiple mediation model in which anxiety/depression and bulimia were mediators between childhood emotional abuse and body mass index (BMI). A set of self-report questionnaires, including the Childhood Trauma Questionnaire (CTQ), Beck Anxiety Inventory, Beck Depression Inventory (BDI), and Eating Disorder Inventory (EDI), was sent out. Clinical data from 37 obese patients (age: 29.65 ± 5.35, body mass index (BMI): 37.59 ± 6.34) and 37 demographically well-matched healthy people with normal body weight (age: 31.35 ± 10.84, BMI: 22.16 ± 3.69) were included in the investigation. We first performed an independent t-test to compare all scales or subscale scores between the two groups. Then, we conducted Pearson correlation analysis to test every two variables’ pairwise correlation. Finally, multiple mediation analysis was performed with BMI as the outcome variable, and childhood emotional abuse as the predictive variable. Pairs of anxiety, bulimia, and depression, bulimia were selected as the mediating variables in different multiple mediation models separately. The results show that the obese group reported higher childhood emotional abuse (*t* = 2.157, *p* = 0.034), worse mood state (anxiety: *t* = 5.466, *p* < 0.001; depression: *t* = 2.220, *p* = 0.030), and higher bulimia (*t* = 3.400, *p* = 0.001) than the healthy control group. Positive correlations were found in every pairwise combination of BMI, childhood emotional abuse, anxiety, and bulimia. Multiple mediation analyses indicate that childhood emotional abuse is positively linked to BMI (*β* = 1.312, 95% CI = 0.482–2.141). The model using anxiety and bulimia as the multiple mediating variables is attested to play roles in the relationship between childhood emotional abuse and obesity (indirect effect = 0.739, 95% CI = 0.261–1.608, 56.33% of the total effect). These findings confirm that childhood emotional abuse contributes to adulthood obesity through the multiple mediating effects of anxiety and bulimia. The present study adds another potential model to facilitate our understanding of the eating psychopathology of obesity.

**Supplementary Information:**

The online version contains supplementary material available at 10.1186/s12889-024-18015-w.

## Introduction

When we consider reasons for overweight and obesity, the early environment is particularly important to address [1]. Eating patterns are often potentially established in childhood and contribute to epigenetic changes and subsequent weight difficulty development [2, 3]. Research has reliably demonstrated associations between childhood maltreatment and body mass index (BMI) [4–6]. Stressful and traumatic childhood abuse experiences highly increase the risk of adulthood overweight [7–10]. The association between childhood traumatic experiences and adulthood obesity has been confirmed [11]. While childhood sexual and physical abuse were also hypothesized to be risk factors in multifactorial models of obesity, the role of emotional abuse gradually gained more attention. Childhood emotional abuse means a sustained, repetitive, inappropriate emotional response to the child’s experience of emotion. Among all kinds of childhood trauma, emotional abuse is highly prevalent and easily occurs since inappropriate emotional responses are instantaneous, less effort is spent by abusers, and the consequence of emotional hurt is insidious and difficult to detect [12]. Childhood emotional abuse is central to understanding the latent effects of child maltreatment, and its potential importance in the etiology of obesity needs further investigation [13].

One possible mediating factor in the relationship between childhood emotional abuse and adult obesity is anxiety. Childhood emotional abuse has a long-term effect on psychiatric performance [14–17]. Specifically, childhood emotional abuse is particularly relevant to the development of anxiety and depression [5, 18–20]. Furthermore, the study showed that obese people are worse in indicators of happiness, perceived mental health, life satisfaction, positive affect, negative affect, optimism, feeling loved and cared for, and depression [21]. Obesity may have long-term implications for mental distress at a clinical level over the adult years [22]. Some studies have shown that obesity is associated with an increase in lifetime anxiety disorders [23, 24]. A meta-analysis review of cross-sectional studies confirmed the association between obesity and anxiety and concluded that obesity was also associated with past-year and lifetime anxiety prevalence [25].

Another possible mediating factor between childhood emotional abuse and obesity is bulimia, which is an eating disorder behavior indicated by a tendency to eat a large amount of food in a short time [26]. Bulimia has a similar meaning to bingeing describing a tendency of excessive or uncontrolled indulgence, especially in food or drink. It is also considered a key symptom of the eating disorder. The literature consistently suggests a close association between bulimia and obesity [2, 27–29]. Bulimia is the most shared direct risk factor for obesity. Distressing psychological states such as anxiety and depression are also likely to increase indulgent food intake, frequent emotional overeating, and bulimia, which are unhealthy eating behaviors that contribute to high rates of obesity [30]. For example, a study has shown that greater attachment anxiety is predictive of a heavier body mass index [31]. A positive correlation has been examined between social anxiety disorder and binge eating frequency [32, 33]. In weight-loss surgery candidates, higher attachment anxiety is associated with a greater incidence of bulimia [34]. In addition, a systematic literature review indicated that some studies demonstrate an association between depression and binge eating disorder, but carefully designed studies are required [35]. While many studies have suggested a negative emotional effect on bulimia, the role of anxiety may be more important for future research [36]. Therefore, we hypothesize that anxiety is a more important mediating factor in the present study.

The prevailing view is that the relationships between anxiety, overeating, and body mass index can be explained in terms of emotion regulation [37]. The emotion regulation system connects to eating behavior by balancing different mental dimensions. Due to early adverse emotional experiences, individuals tend to be hyperactivated to potentially upsetting/stressful negative social cues. They are relatively poor at managing their emotions and thus more likely to be anxious [38, 39]. Therefore, to ‘soothe’ themselves, some anxious individuals rely on external sources of affect regulation such as food, while others may choose to rely on smoking, substance misuse, etc. [40]. Studies have proven that anxiety is specifically related to emotional eating among weight-loss surgery candidates [41]. Obesity seems to involve higher emotional dysregulation than normal weight conditions [42]. Emotion regulation is essential in the relationship between anxiety and bulimia, as it could represent a risk factor for the worsening of problems related to overeating and excessive body weight [34]. Thus, the importance of the emotion regulation process has been considered in the success of weight-loss treatment and could provide significant clinical information and therefore be part of the obesity diagnostic criteria and therapeutic program [43].

The etiology and maintenance of obesity have been substantially advanced. Based on the preproved potential connections between childhood trauma, adverse mood state, and bulimia, we hypothesize that there are explicit multiple mediation models linking all the possible variables of childhood emotional abuse, anxiety/depression, and bulimia and obesity (body mass index). Here, we sampled some weight-loss surgery candidates at hospitals and well-matched healthy controls to establish multiple mediation models to (1) add an approval of the association between childhood emotional abuse and obesity; (2) reveal the potential pathway between childhood emotional abuse and obesity, in which anxiety/depression could serve as intermediate factors; and (3) compare the model fit to test whether anxiety/depression play an equal main mediating effect in this relationship. The multiple mediation models will help clinicians continue to disentangle interactions of these factors to further facilitate our understanding of eating psychopathology.

## Method

### Participants

From September 2020 to January 2021, obese patients who were going to have weight-loss surgery at the Department of Bariatric & Metabolic Surgery, Shanghai Jiao Tong University Affiliated Sixth People’s Hospital, were informed about the present study. Inclusion criteria for the obesity are (1) meet the indication of bariatric surgery: BMI ≥ 32.5 or BMI between 32.5 to 27.5 with comorbidity of metabolic syndromes; (2) aged above 18 years old; (3) able to read and understand the description of each item of the questionnaire; and (4) voluntarily participated in the survey and signed the informed consent form were invited to participate in the present study to answer a set of clinical scales. Department of Bariatric & Metabolic Surgery, Shanghai Jiao Tong University Affiliated Sixth People’s Hospital handed out healthy control recruitment advertisements in the nearby community. Citizens who were interested in participating in the research would contact the experimenters directly. Inclusion criteria for the healthy controls are (1) with normal figures and do not meet the indication of bariatric surgery; (2) without any eating disorders; (3) demographically matched with obese patients’ characteristics (with similar means of age, education years and similar gender ratio); (4) voluntarily participated in the survey and signed the informed consent. Both participants in obesity and healthy control were first interviewed by a professional psychiatrist. Participants examined with psychiatric disorders would be excluded from the research. The present study was approved by the Ethics Committee of Shanghai Sixth People’s Hospital, NO 2020–219-(1). All procedures followed the Declaration of Helsinki. Eventually, we analyzed the clinical data from 37 obese patients and 37 healthy people.

### Clinical measurement

Basic demographic information (age, years of education, height, weight) and a series of clinical scales (Childhood Trauma Questionnaire, CTQ; Beck Anxiety Inventory, BAI; Beck Depression Inventory, BDI; and Eating Disorders Inventory, EDI) were collected. Body mass index (BMI, equal to weight (in kilograms) divided by height (in meters) squared) was calculated to describe the severity of obesity. All demographic information and clinical scales were presented to participants in the form of an online questionnaire designed by a professional psychologist. Participants answered all online questionnaires using their cell phones in the examination room with the supervision of experimenters. Demographic information on height and weight was self-reported by participants, experimenters would invite them to use the height and weight gauge in the examination room to measure these indexes when they are not sure.

The Childhood Trauma Questionnaire (CTQ) [44] is designed for adolescents and adults to obtain a brief, reliable, and valid assessment of traumatic experiences in childhood [45, 46]. It assesses the incidents of abuse and neglect in childhood, including physical abuse, emotional abuse, sexual abuse, emotional neglect, and physical neglect [47]. The total Cronbach’s α of the Chinese version of the CTQ is 0.73 [44]. The CTQ has 28 items, including a minimization–denial subscale of 3 items, and each item adopts a 5-point Likert score from 1 “never” to 5 “always” according to the frequency of the experiences that occurred [46]. Scores for each of the categories include 5 items, ranging from 5 to 25. A higher CTQ score indicates more severe childhood trauma. The total CTQ Cronbach’s α of the present sample is 0.623, and the Cronbach’s α values of the physical abuse, emotional abuse, sexual abuse, emotional neglect, and physical neglect subscales are 0.648, 0.793, 0.641, 0.746, and 0.462, respectively.

The Beck Anxiety Inventory [48, 49] assesses the severity of generalized anxiety symptoms [50]. It has good reliability, validity, internal consistency, and convergence [51, 52]. The total Cronbach’s α of the Chinese version of the BAI is 0.95 [49]. The BAI has 21 items, with each response based on a 4-point Likert scale ranging from 0 “not at all” to 3 “severely”. A higher score indicates greater anxiety severity. The total BAI Cronbach’s α of the present sample is 0.920.

The Beck Depression Inventory (BDI) [53] version 2 is a widely used clinical instrument to evaluate depression severity in normal populations [54–56]. It has good reliability and validity [55]. The total Cronbach’s α of the Chinese version of the BDI is 0.94 [53]. The BDI has 21 items, and each item consists of four self-evaluative statements scored from 0 to 3, with an increasing score indicating greater depression severity. The total BDI Cronbach’s α of the present sample is 0.901.

The Eating Disorder Inventory version 2 (EDI-2) measures eating disorder symptoms and the cognitive and behavioral characteristics of anorexia nervosa and bulimia [57]. The EDI-2 contains 91 items, with each response based on a 6-point Likert scale ranging from 0 “never” to 5 “always” [58]. It consists of 11 subscales. Three subscales measure the primary eating disorder symptoms: (1) drive for thinness, (2) bulimia and (3) body dissatisfaction. Eight subscales measure correlated psychological traits: (4) ineffectiveness, (5) perfectionism, (6) interpersonal distrust, (7) interceptive awareness, (8) maturity fear, (9) asceticism, (10) impulse regulation, and (11) social insecurity. The total EDI-2 Cronbach’s α of the present sample is 0.925. The Cronbach’s α subscales were drive for thinness, 0.767; bulimia, 0.840; body dissatisfaction, 0.823; ineffectiveness, 0.823; perfectionism, 0.667; interpersonal distrust, 0.742; interceptive awareness, 0.706; maturity fear, 0.477; asceticism, 0.599; impulse regulation, 0.737; and social insecurity, 0.767.

### Statistical analysis

To test common method biases, we performed Harman’s single-factor test [59]. Unrotated factor analysis revealed that the eigenvalues of 27 factors were > 1. The first factor explained only 26.42% of the variance, which was much lower than the threshold of 40%. The results indicated the absence of severe common method biases in the investigation.

Statistical analysis was carried out with SPSS 21. To narrow down the possible appropriate variables for the multiple mediation model. We first performed the independent t-test to make a comparison between the obesity group and healthy controls (sex distribution comparison was verified through χ^2^-test). Then, the factors that had a significant between-group difference were selected for Pearson correlation analysis to further verify the possibility of building the multiple mediation model. The significance level alpha was 0.05 (two-tailed).

Multiple mediation analysis was conducted by the PROCESS macro in SPSS, as developed by Hayes [60]. Multiple mediation models can estimate a specific indirect effect to describe how the independent variable leads the dependent variable through intermediate factors [61]. A bootstrap method was adopted to construct a 95% confidence interval for significance testing of mediating effects.

## Results

### Difference of demographic information and clinical measurements between patients and healthy controls

The demographic information and clinical variables of the obese patients and the healthy control group are shown in Table 1. There was no significant difference in gender distribution, age, or education years between the two groups.

**Table 1:** Independent t-test of demographic information and clinical measurements between obesity and healthy control group

	Obesity *n* = 37 **(Mean** ± **SD)**	Healthy control *n* = 37 **(Mean** ± **SD)**	*df*	*t/*χ^2^	*P*	*Effect size (****φ, Cohen’s d)***
**Demographic information**						
Gender (Female/ Male)	27/10	28/9	1	0.071	0.790	0.001
Age	29.650 ± 5.345	31.350 ± 10.840	52.527	-0.857	0.395	-0.199
Education years	14.140 ± 2.605	15.220 ± 4.217	72	-1.327	0.190	-0.308
BMI	37.592 ± 6.336	22.156 ± 3.687	57.867	12.808***	< 0.001	2.978
**Mood state**						
Anxiety (BAI)	9.810 ± 8.356	1.950 ± 2.603	68.487	5.466***	< 0.001	1.270
Depression (BDI)	11.300 ± 9.279	6.970 ± 7.369	72	2.220*	0.030	0.517
**Childhood Trauma Questionnaire (CTQ)**						
Emotional abuse	7.110 ± 2.558	6.000 ± 1.795	72	2.157*	0.034	0.502
Physical abuse	5.700 ± 1.648	5.160 ± 0.688	48.175	1.842	0.072	0.428
Sexual abuse	5.220 ± 0.750	5.220 ± 0.712	72	0.000	1.000	0
Emotional neglect	18.650 ± 1.783	18.840 ± 1.708	72	-0.466	0.643	-0.144
Physical neglect	7.950 ± 2.877	8.220 ± 2.849	72	-0.406	0.686	-0.094
**Eating disorder Inventory (EDI)**						
Drive for thinness	1.154 ± 0.587	0.537 ± 0.490	72	4.914***	< 0.001	1.141
Bulimia	0.514 ± 0.533	0.158 ± 0.346	61.806	3.400***	0.001	0.792
Body dissatisfaction	1.955 ± 0.411	1.330 ± 0.488	72	5.953***	0.000	1.385
Ineffectiveness	0.838 ± 0.519	0.722 ± 0.353	72	0.368	0.714	0.261
Perfectionism	0.748 ± 0.595	0.928 ± 0.563	72	-1.337	0.185	-0.311
Interpersonal distrust	1.236 ± 0.657	1.290 ± 0.645	72	-0.367	0.722	-0.083
Interoceptive awareness	0.468 ± 0.324	0.397 ± 0.309	72	0.955	0.343	0.224
Maturity fear	1.497 ± 0.459	1.368 ± 0.453	72	1.212	0.230	0.282
Asceticism	0.470 ± 0.286	0.446 ± 0.348	72	0.319	0.750	0.075
Impulse regulation	0.381 ± 0.384	0.189 ± 0.338	72	2.278*	0.026	0.531
Social insecurity	1.298 ± 0.567	1.318 ± 0.655	65.487	-0.129	0.897	-0.033

*BMI* Body Mass Index, *BDI* Beck Depression Inventory, *BAI* Beck Anxiety Inventory

^*^p < 0.05, **p < 0.01, ***p < 0.001

**Fig. 1:**
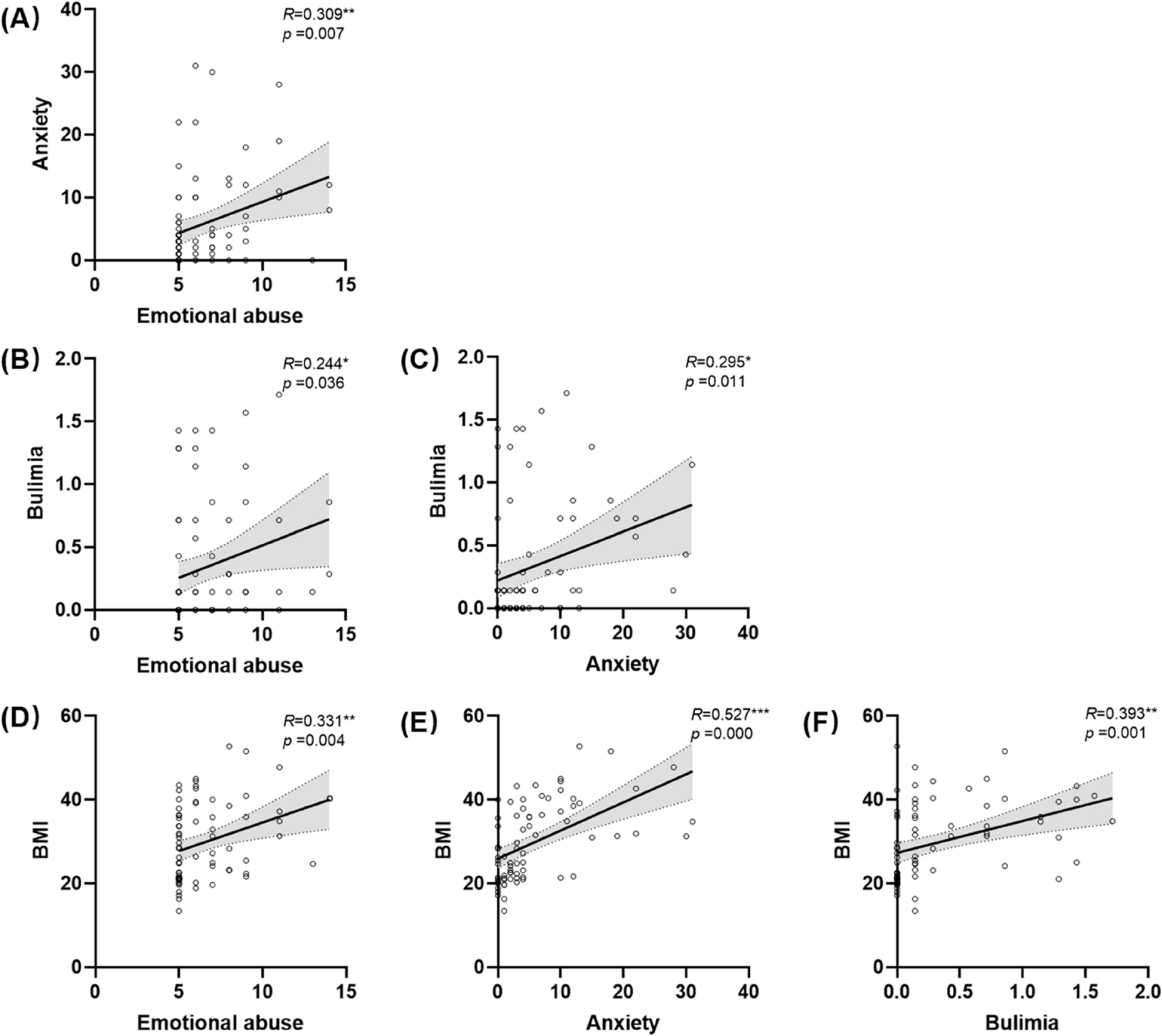
Pair correlation diagram of Pearsons’ correlation analysis among emotional abuse, anxiety, bulimia, and BMI. BMI: Body Mass Index. The *P*-values of the Pearson correlation analysis have not been corrected

Obese patients were more anxious (*t* = 5.466, *p* < 0.001, df = 68.487, Cohen’s d = 1.270) and depressive (*t* = 2.220, *p* = 0.030, df = 72, Cohen’s d = 0.517), experienced more childhood emotional abuse (*t* = 2.157, *p* = 0.034, df = 72, Cohen’s d = 0.502) and had a higher intensity of drive for thinness (*t* = 4.914, *p* < 0.001, df = 72, Cohen’s d = 1.141), bulimia (*t* = 3.400, *p* = 0.001, df = 61.806, Cohen’s d = 0.792), body dissatisfaction (*t* = 5.953, *p* < 0.001, df = 72, Cohen’s d = 1.385) and impulse regulation (*t* = 2.278, *p* = 0.026, df = 72, Cohen’s d = 0.531) than healthy controls. Significant correlations existed among these variables (except BMI and BDI, emotional abuse, and impulse regulation). The correlation coefficients are shown in Table 2 (The *p*-values of the Pearson correlation analysis have not been corrected). The variable of bulimia, a tendency to overeat a large amount of food without control as the primary reason for gaining weight, was selected with variables of childhood emotional abuse, anxiety/depression, and BMI to build the multiple mediation model. Variables of drive for thinness, body dissatisfaction, describing expectations of losing weight which would highly increase after people getting weight, thus contribute far less than variable of bulimia. Variables of impulse regulation inadequately explain obesity compared with the variable of bulimia. A variable of depression was excluded because it has no significant correlation with BMI. Figure 1 only presents the overall positive correlations of pairwise combinations of BMI, anxiety, childhood emotional abuse, and bulimia which mutually influence each other from a clinical perspective.

**Table 2:** Correlation coefficients of the main variables with significant differences between groups

	BMI	BAI	BDI	Childhood emotional abuse	Drive for thinness	Bulimia	Body dissatisfaction
BAI	0.527***	-					
BDI	0.218	0.593**	-				
Childhood emotional abuse	0.331**	0.309**	0.415**	-			
Drive for thinness	0.428**	0.324**	0.330**	0.258*	-		
Bulimia	0.393**	0.295*	0.282*	0.244*	0.492*	-	
Body dissatisfaction	0.617**	0.400**	0.296*	0.324**	0.572**	0.431**	-
Impulse regulation	0.251*	0.553**	0.445**	0.103	0.359**	0.475**	0.277*

The *P*-values of the Pearson correlation analysis have not been corrected

*BMI* Body Mass Index, *BDI* Beck Depression Inventory, *BAI* Beck Anxiety Inventory

^*^p < 0.05, **p < 0.01, ***p < 0.001

### Multiple mediation effect testing

**Fig. 2:**
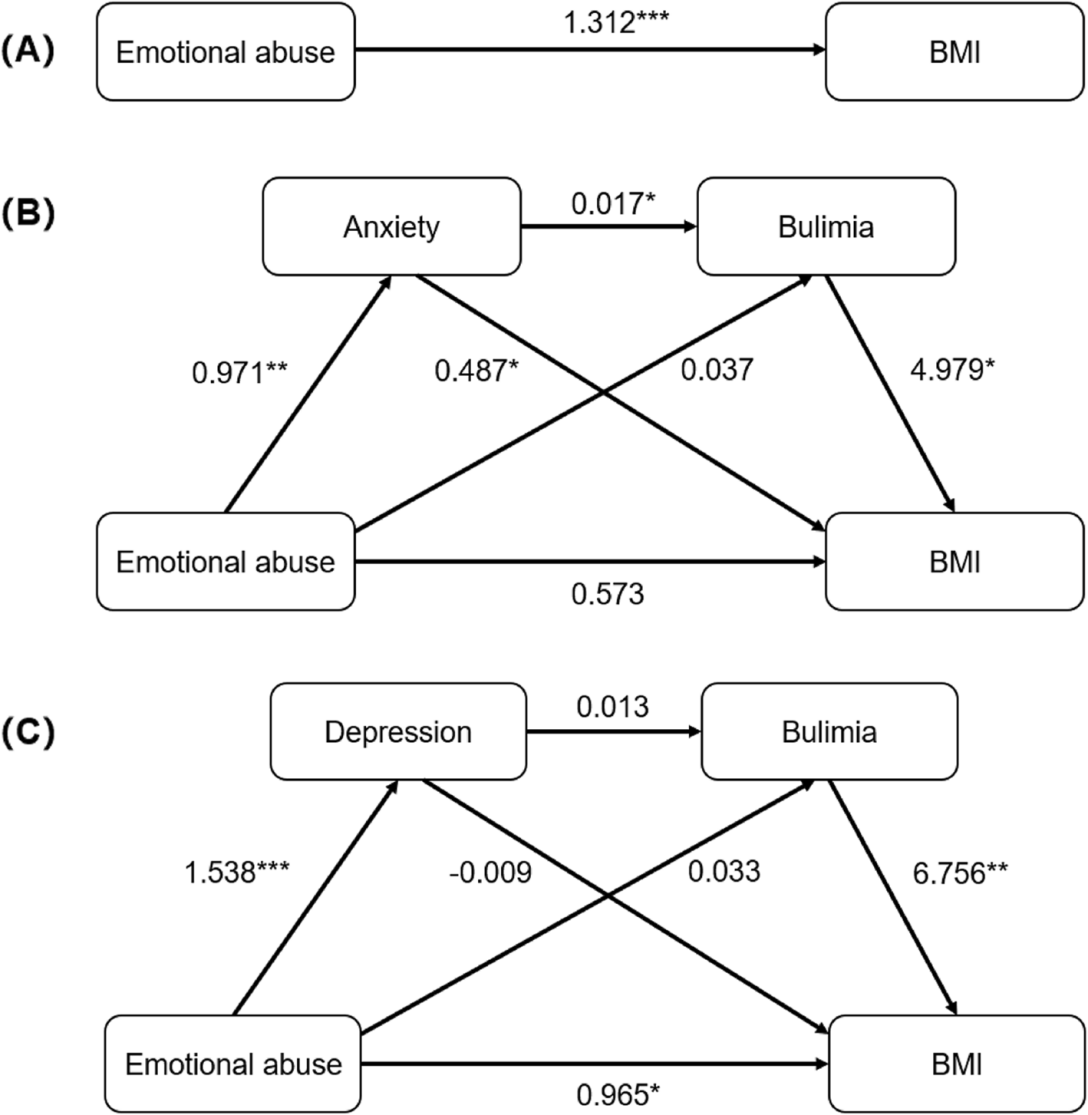
Multiple mediation pathway models. **A** Total effects of emotional abuse on BMI; **B** Model1: Anxiety and bulimia as multiple mediate variables; **C** Model2: Depression and bulimia as multiple mediate variables. BMI: Body Mass Index. *p < 0.05, **p < 0.01, ***p < 0.001

To ensure better external and ecological validity of this multiple mediation model, we performed mediation effect testing with the original data. Controlling for demographic information (age and education years), the PROCESS macro was used to verify the multiple mediating roles of anxiety, depression, and bulimia in the relationship between childhood emotional abuse and BMI. Figure 2 shows the mediation pathway models.

We used the bootstrap method to test the significance of the mediating effects, in which the sampling process was repeated 1000 times to calculate the 95% confidence interval. Table 3 presents the results of the mediation analysis. Table 4 present the results of the multiple mediation analysis. The mediation pathway was significant if the 95% confidence interval of the path coefficient did not contain 0.

**Table 3:** Bootstrap analysis of multiple mediation effects

Regression model		Goodness-of-fit indices			Regression coefficient and significance	
Outcome variable	Predictor variable	*R*	*R*^*2*^	*F*	β	*t*
**Total effects**						
BMI		0.391	0.153	4.721		
	Emotional abuse				1.312	3.154***
**Model1: Anxiety as one of the multiple mediate variables**						
Anxiety		0.391	0.153	4.069		
	Emotional abuse				0.971	2.644**
Bulimia		0.360	0.129	2.02		
	Anxiety				0.017	2.029*
	Emotional abuse				0.037	1.350
BMI		0.617	0.380	6.604		
	Anxiety				0.487	2.511*
	Bulimia				4.979	2.247*
	Emotional abuse				0.573	1.432
**Model2: Depression as one of the multiple mediate variables**						
Depression		0.436	0.190	6.534		
	Emotional abuse				1.538	3.577***
Bulimia		0.346	0.120	1.688		
	Depression				0.013	1.568
	Emotional abuse				0.033	1.286
BMI		0.514	0.264	5.291		
	Depression				-0.009	-0.081
	Bulimia				6.756	3.130**
	Emotional abuse				0.965	2.017*

Bootstrap analysis of multiple mediation effects

*BMI* Body Mass Index

^*^p < 0.05, **p < 0.01, ***p < 0.001

**Table 4:** Results of the multiple mediation analysis

	Effect size	SE	Percentage of total effects	95% CI	
				Lower limit	Upper limit
**Total effects**					
Emotional abuse → BMI	1.312	0.416	100.00%	0.482	2.141
**Model1: Anxiety and bulimia as multiple mediate variables**					
Indirect effects	0.739	0.356	56.33%	0.261	1.608
Pathway1: Emotional abuse → Anxiety → BMI	0.473	0.293	36.03%	0.126	1.287
Pathway2: Emotional abuse → Bulimia → BMI	0.185	0.185	14.06%	-0.026	0.700
Pathway3: Emotional abuse → Anxiety → Bulimia → BMI	0.082	0.052	6.24%	0.018	0.283
**Model2: Depression and bulimia as multiple mediate variables**					
Indirect effects	0.347	0.282	26.45%	-0.106	0.996
Pathway4: Emotional abuse → Depression → BMI	-0.014	0.189	-1.07%	-0.376	0.396
Pathway5: Emotional abuse → Bulimia → BMI	0.222	0.208	16.92%	-0.037	0.784
Pathway6: Emotional abuse → Depression → Bulimia → BMI	0.140	0.102	10.67%	0.003	0.423

*BMI* Body Mass Index

In both models, the total effect (emotional abuse → BMI) was significant (effect size = 1.312, 95% CI = 0.482–2.141), with significant direct effect (β = 1.321***). For Model 1, in which anxiety and bulimia were the multiple mediating variables, the total indirect effects accounted for 56.33% of the total effects. Specifically, the effect of the emotional abuse → anxiety → bulimia → BMI pathway was significant (effect size = 0.082, 95% CI = 0.018–0.283), accounting for 6.24% of the total effects. The direct effects of emotional abuse → anxiety (β = 0.971**), anxiety → bulimia (β = 0.017*), bulimia → BMI (β = 4.979*) within this pathway were significant. The effect of the emotional abuse → anxiety → BMI pathway was also significant (effect size = 0.473, 95% CI = 0.0126–1.608), accounting for 36.03% of the total effects. The direct effects of anxiety → BMI (β = 0.487*) within this pathway was significant. However, the effect of emotional abuse → bulimia → BMI was not significant.

For Model 2, in which depression and bulimia were the multiple mediating variables, the total indirect effects accounted for 26.45% of the total effects. Specifically, the effect of the emotional abuse → depression → bulimia → BMI pathway was significant (effect size = 0.140, 95% CI = 0.003–0.423), accounting for 10.67% of the total effects. The direct effects of emotional abuse → depression (β = 1.538***), bulimia → BMI (β = 6.756**) within this pathway were significant, while the direct effect of depression → bulimia (β = 0.013) was not significant. However, the emotional abuse → depression → BMI pathway was not significant. The direct effects of depression → BMI (β = -0.009) within this pathway was not significant. The emotional abuse → bulimia → BMI pathway was not significant either.

When comparing the multiple mediation results of these two models (Table 4), we found that (1) the indirect effects of Model 1 were much higher than those of Model 2; (2) for both models, the emotional abuse → anxiety/depression → bulimia → BMI pathway effect was significant, but the emotional abuse → bulimia → BMI pathway effect was not; (3) the emotional abuse → anxiety → BMI pathway of Model 1 was significant, while the emotional abuse → depression → BMI pathway of Model 2 was not. (4) The direct effect of anxiety → BMI and anxiety → BMI were significant, while the direct effect of depression → bulimia and depression → BMI were not significant. These results indicated that anxiety plays an important role in the multiple mediation model.

## Discussion

The present study proves that childhood emotional abuse is associated with obesity and establishes a multiple mediation model in which anxiety/depression and bulimia play multiple mediating roles in the relationship between childhood emotional abuse and adult obesity. Our study found three main results: (1) there was a positive association between childhood emotional abuse and adult obesity, (2) childhood emotional abuse significantly influenced adult obesity through the anxiety–bulimia pathway, and (3) childhood emotional abuse did not significantly influence adult obesity through the depression–bulimia pathway. These findings add to the literature and may enhance public awareness of the relationships between early life trauma, mental health, and unhealthy eating patterns while providing a better understanding of obesity as it relates to childhood emotional abuse and anxiety.

Among various types of childhood maltreatment, emotional abuse of obesity came to be significantly higher than the healthy control, while others did not appear to be significantly different. Emotional abuse in childhood and obesity in adulthood is a cliché. Child emotional abuse is a universal problem affecting the lives of millions of children worldwide. The international prevalence of emotional abuse may be 36.30% [62]. Individuals who suffered abuse had a 93% increased risk of BMI ≥ 40 kg/m^2^ [63]. In our sample, only the obese patients reported significantly higher childhood emotional abuse. This finding adds additional evidence of the easy occurrence and high prevalence of emotional abuse among obese individuals in their childhood. Childhood emotional abuse is now thought to be more prevalent than other forms of abuse due to its insidious threat to the adaptation of victims [64]. In China, emotional abuse may be one of the most prevalent types of maltreatment with a much higher prevalence, a study indicated that the prevalence of emotional abuse among adolescents was 51.40% [65–67].

Anxiety is thought to be an important mediator of emotional abuse in childhood and obesity in adulthood. Childhood emotional abuse, involving a repeated pattern of caregiver behavior or a serious incident, transmits negative information to the child that he or she is worthless, flawed, unloved, unwanted, endangered, or only of value in meeting another’s needs [68]. Spurning, intimidating and terrorizing, confining and isolating, exploiting and corrupting, denigrating emotional needs, and neglecting health needs manifest negative impacts on a child’s emotions and daily functionality and seriously undermine a child’s future adaptation [69–71]. Mechanisms linking emotional abuse with anxiety include maladaptive self-experience, such as with resilience and self-esteem. Psychological maltreatment reduces children’s psychological resilience, which is a positive resource adolescents can utilize to manage stressful challenges [72–74]. Emotional abuse causes low self-esteem, including negative evaluations of oneself [75, 76]. Undeveloped self-experience highly increases susceptibility to developing anxiety and depression by causing a series of difficulties identifying emotions and emotional awareness and is more likely to induce anxiety in adulthood [77]. These findings may explain the evidence of a higher prevalence of lifetime diagnosed anxiety in obesity [78].

Bulimia symptoms are considered emotionally induced psychosomatic symptoms, and the tendency to bulimia in obese people is closely related to obesity. To further prove the connection sequence of anxiety and bulimia, we built and examined a model in which bulimia was the first multiple mediating variable and anxiety was the second multiple mediating variable (SFig. 1). The model fitting result was less satisfactory than the original model (anxiety was the first multiple mediator variable; see Supplementary material: the regression coefficient of emotional abuse → bulimia → anxiety/depression → BMI was not significant, STables 1 and 2). According to previous studies, anxiety disorders commonly have an onset in childhood and frequently exist before eating disorders [79, 80]. The model fitting and clinical evidence indicated that childhood emotional abuse might primarily lead to anxious traits. The recurring anxious emotional state triggers more bulimic behavior and further leads to obesity.

Depression is thought to be a mediating factor outside of anxiety between childhood emotional abuse and adult obesity. Our study described one indirect pathway of childhood emotional abuse contributing to obesity and demonstrated that anxiety plays an important mediating role in this relationship. This result provides a new perspective for treating obese patients with adverse early life events. Beyond bariatric surgery, psychological intervention is also helpful in reducing the influence of predisposing pathogenic factors. In future treatment, it would be beneficial to offer obese patients psychological therapy to reduce their anxiety and bulimic behavior. Anxiety and obesity are the two most common related health problems [81]. It is highly possible that anxiety disorders would lead to weight gain. For stressed individuals, the dysregulation of the hypothalamic–pituitary–adrenal axis contributes to subsequent getting weight [82, 83]. Symptoms of anxiety stimulate a craving for high-sugar and high-fat foods [84–86]. Anxiety-related chronic conditions might also have an influence on functional health, which may cause physical inactivity leading to excess weight. Previous studies also reported that anxiety is strongly associated with binge eating and emotional eating [33]. Obese patients eat more when they feel anxious, and the aroused effect is significantly reduced after gluttonous eating [87]. Therefore, anxiety is a critical factor in the childhood emotional abuse–obesity relationship, since the high likelihood of an anxious emotional state triggers bulimic behavior. In contrast, the connection between depression and bulimia is ambiguous. A depressive state does not always increase eating. In a sample of depressed patients, only 14% indicated an increase in appetite, while in 66%, appetite decreased, and in 20%, it showed no change [88]. Bradley M. Appelhans et al. reported that more severe depression is associated with more inferior diet quality [89]. For these reasons, we believe that anxiety plays a vital role in leading these obese patients to perform more bulimic behavior, which could release their anxious impulses but cause excessive fat accumulation.

Many studies have suggested that unhealthy eating habits, including overeating and bulimia, could be the result of a failure to attempt to regulate negative emotions [90–92]. Lack of emotion regulation could lead to a breakdown in the autoregulation of other personal areas, including those linked to the control of eating behavior [93, 94]. Emotion regulation is the ability to regulate one’s own positive or negative emotions to diminish, attenuate, maintain, or amplify their content [95]. For obese patients, when their emotions are dysregulated, maladaptive behaviors can be adopted to encourage them to overeat in response to anxiety [94, 96]. The emotionally driven eating model explains inappropriate eating behaviors by suggesting dysfunctional emotional and cognitive processing as causes of overeating [97]. The deficit in the regulation of eating behavior can be attributed to a failure in emotional regulation that can lead to overeating behavior to compensate for an inability to employ proper cognitive strategies to avoid a negative emotional state [92, 98]. Therefore, the ability to regulate anxiety or dysphoric mood is associated with binge eating and emotional eating in overweight individuals and has been considered a critical target to reduce excess body weight [33, 99, 100]. The clinical implications of the proposed multiple mediation models in this research strengthen the obesity treatment idea that improving obese patients’ emotion regulation ability is an effective target to rectify unhealthy bulimia behavior. Some pilot randomized controlled trial studies have achieved some therapeutic effects. For example, Berking and Whitley developed emotion regulation training (EuREKA), which is an innovative intervention program for children and adolescents that aims to examine the effectiveness of emotion regulation training when combined with a multidisciplinary obesity treatment in inpatient-treated 10- to 14-year-old youngsters [101, 102]. Obese youngsters of the EuREKA program exhibited less emotional eating behavior and improved weight loss and weight-loss maintenance, causally proving that emotion regulation intervention can be applied in clinical practice [103].

Some limitations of the present study should be noted. First, the obese participants recruited in the sample are patients seeking bariatric surgery in the hospital, which only presents a subpopulation of the obese. Compared to the obese, candidates for bariatric surgery display advantageous personality features and lower rates of psychopathology [104]. Second, its sample size is relatively small. Future studies need to collect a larger sample to make a firmer conclusion. Third, the present study is a cross-sectional investigation. All participants estimated their childhood experiences based on their retrospective memory. Longitudinal designs and interventional experiments should be adopted in future studies to reveal sequential causality. Finally, the data collection was based on the self-report questionnaire, which inevitably led to reported biases even though we strictly controlled the response quality. More objective indicators of neuroimaging are necessary. Childhood maltreatment reduces left-side hippocampal volumes [105–107] and the functional integrity of white matter tracts [108, 109]. Increased insula activation is involved in the neurological processing of food-related stimuli [110, 111]. Diminished frontostriatal activity contributes broadly to emotion, motivation, and movement processes and, importantly, is thought to underlie self-regulatory control [112]. More functional connections between related brain areas need to be confirmed to further substantiate the multiple mediation models of the present study. More evidence from random clinical trial research about emotional regulation training in obesity treatment will also make the current conclusion more stable.

In conclusion, obese patients experienced more childhood emotional abuse and were more anxious, depressive, and bulimic than healthy people. Childhood emotional abuse may contribute to adulthood obesity, potentially mediated by anxiety and bulimia. In obesity treatment, psychological interventions such as emotion regulation training would be helpful to reduce anxious emotions and thus decrease bulimic behavior.

### Supplementary Information

**Supplementary material 1.**

## Data Availability

The datasets used and analysed during the current study available from the corresponding author on reasonable request.
