# Supplementary material for: Multiple mediation of the association between childhood emotional abuse and adult obesity by anxiety and bulimia – a sample from bariatric surgery candidates and healthy controls
Source: BMC Public Health. 2024 Mar 1;24:653. doi: 10.1186/s12889-024-18015-w (PMC10905949; doi:10.1186/s12889-024-18015-w)
Supplement: Supplementary file 1 — Supplementary material 1. [file 12889_2024_18015_MOESM1_ESM.docx]

**Supplementary materials：Possible competition model**

**SFigure1: Multiple mediation pathway models**


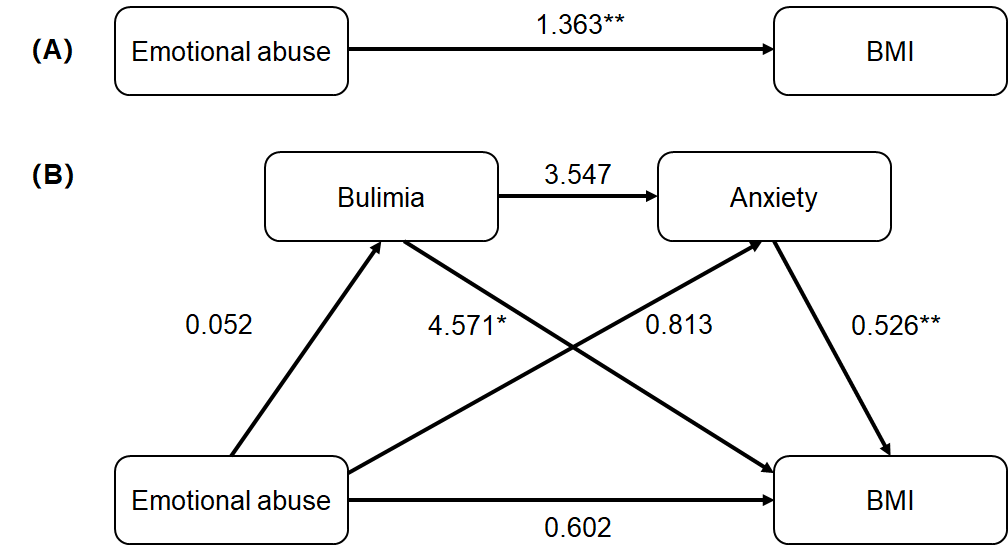


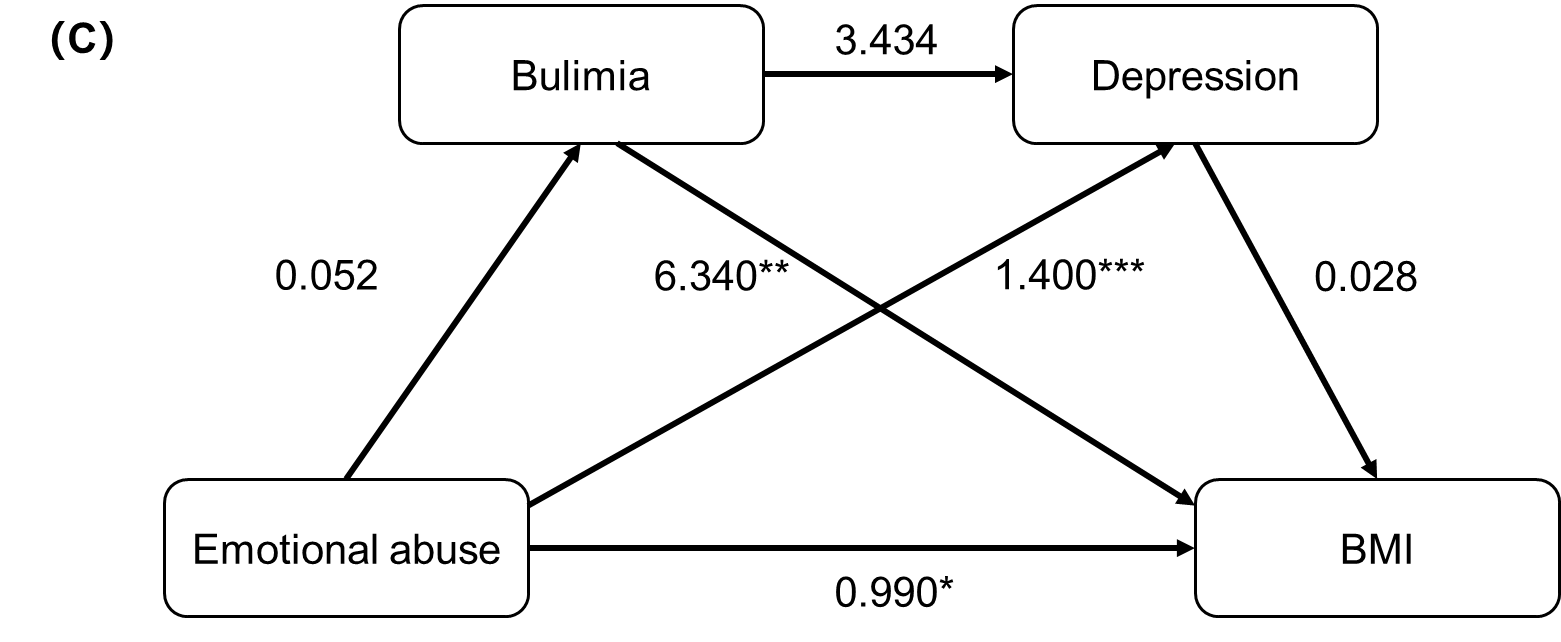


SFigure1: Multiple mediation pathway models. (A) Total effects of emotional abuse on BMI; (B) Model1: Bulimia and anxiety as multiple mediate variables; (C) Model2: Bulimia and depression as multiple mediate variables. BMI: Body Mass Index. **p*<0.05, ***p*<0.01, ****p*<0.001.

| Regression model | | Goodness-of-fit indices | | | Regression coefficient and significance | |
| --- | --- | --- | --- | --- | --- | --- |
| Outcome variable | Predictor variable | *R* | *R^2^* | *F* | *β* | t |
| **Total effects** | | | | | | |
| BMI |  | 0.331 | 0.110 | 9.793 |  |  |
|  | Emotional abuse |  |  |  | 1.363 | 3.129** |
| **Model1: Anxiety as one of the multiple mediate variables** | | | | | | |
| Bulimia |  | 0.244 | 0.059 | 3.614 |  |  |
|  | Emotional abuse |  |  |  | 0.052 | 1.901 |
| Anxiety |  | 0.383 | 0.146 | 6.764 |  |  |
|  | Bulimia |  |  |  | 3.547 | 1.624 |
|  | Emotional abuse |  |  |  | 0.813 | 1.811 |
| BMI |  | 0.599 | 0.359 | 10.758 |  |  |
|  | Bulimia |  |  |  | 4.571 | 2.055* |
|  | Anxiety |  |  |  | 0.526 | 2.861** |
|  | Emotional abuse |  |  |  | 0.602 | 1.534 |
| **Model2: Depression as one of the multiple mediate variables** | | | | | | |
| Bulimia |  | 0.244 | 0.059 | 3.614 |  |  |
|  | Emotional abuse |  |  |  | 0.052 | 1.901 |
| Depression |  | 0.455 | 0.207 | 10.847 |  |  |
|  | Bulimia |  |  |  | 3.434 | 1.492 |
|  | Emotional abuse |  |  |  | 1.400 | 3.513*** |
| BMI |  | 0.462 | 0.214 | 7.632 |  |  |
|  | Bulimia |  |  |  | 6.340 | 2.913** |
|  | Depression |  |  |  | 0.028 | 0.244 |
|  | Emotional abuse |  |  |  | 0.990 | 2.018* |

# STable1: Bootstrap analysis of multiple mediation effects

STable1: Bootstrap analysis of multiple mediation effects. BMI: Body Mass Index. **p*<0.05, ***p*<0.01, ****p*<0.001

# STable2：Results of the multiple mediation analysis

|  | Effect size | SE | Percentage of total effects | 95% CI | |
| --- | --- | --- | --- | --- | --- |
|  |  |  |  | Lower limit | Upper limit |
| **Total effects** |  |  |  |  |  |
| Emotional abuse→BMI | 1.312 | 0.416 | 100.00% | 0.482 | 2.141 |
| **Model1: Anxiety and bulimia as multiple mediate variables** | | | | | |
| Indirect effects | 0.761 | 0.388 | 55.83% | 0.272 | 1.743 |
| Pathway1: Emotional abuse→Bulimia→BMI | 0.237 | 0.189 | 17.39% | 0.011 | 0.832 |
| Pathway2: Emotional abuse→Anxiety→BMI | 0.428 | 0.331 | 31.40% | 0.043 | 1.265 |
| Pathway3: Emotional abuse→ Bulimia→Anxiety→BMI | 0.097 | 0.076 | 7.12% | 0.007 | 0.34 |
| **Model2: Depression and bulimia as multiple mediate variables** | | | | | |
| Indirect effects | 0.373 | 0.276 | 27.37% | -0.057 | 1.109 |
| Pathway4: Emotional abuse→Bulimia→BMI | 0.328 | 0.214 | 24.06% | 0.039 | 0.906 |
| Pathway5: Emotional abuse→Depression→BMI | 0.04 | 0.178 | 2.93% | -0.319 | 0.409 |
| Pathway6: Emotional abuse→ Bulimia→Depression→BMI | 0.005 | 0.028 | 0.37% | -0.023 | 0.121 |

STable2: Results of the multiple mediation analysis. BMI: Body Mass Index.
